# *Henipaviruses*—A constant threat to livestock and humans

**DOI:** 10.1371/journal.pntd.0010157

**Published:** 2022-02-18

**Authors:** Susann Kummer, Denise-Carina Kranz

**Affiliations:** Center for Biological Threats and Special Pathogens, Robert Koch Institute, Berlin, Germany; NIAID Integrated Research Facility, UNITED STATES

## Abstract

In this review, we highlight the risk to livestock and humans from infections with *henipaviruses*, which belong to the virus family Paramyxoviridae. We provide a comprehensive overview of documented outbreaks of Nipah and Hendra virus infections affecting livestock and humans and assess the burden on the economy and health systems. In an increasingly globalized and interconnected world, attention must be paid to emerging viruses and infectious diseases, as transmission routes can be rapid and worldwide.

## Emergence of infectious diseases

Infectious disease outbreaks have devastated the human population throughout history. The Black Death (1347 to 1351, 25 million deaths), smallpox (1520 to 1979, 56 million deaths), and/or Spanish flu (1918 to 1920, 50 to 100 million deaths) were serious and devastating pandemics in the past [1]. Nowadays, HIV/AIDS, cholera (latest outbreak 2018), or the ongoing Coronavirus Disease 2019 (COVID-19) pandemic are serious threats to human populations, causing significant economic and health burdens with high morbidity and mortality rates and bringing them into the focus of government authorities as a global concern [2].

Emerging diseases are per definition evoked by pathogens entering a new geographic area, expand their host range by transmission, for example, from wildlife to domesticated animals, and harbor a great potential to increase in number in the near future. The pandemic spread of emerging diseases is the result of a combination and interplay of manifold processes like the ongoing globalization (increased commercial air travel and trade), the change of lifestyles and urbanization leading to a massive deforestation and, thus, rerouting of wildlife migration patterns and closer contact of wildlife with domestic animals (farming), which accelerate the occurrence and circulation of newly appearing microbial agents [1]. Various factors influence the emergence of disease outbreaks, for example, environmental conditions or public health infrastructure. Among these factors, the genetic plasticity of the infectious agent plays an important role. Depending on the potential of the individual pathogen to evolve and adapt to ecological niches and new hosts, the likelihood increases that it can spread and facilitate its own transmission, which could lead to a global spread of the pathogen [2].

**Fig 1:**
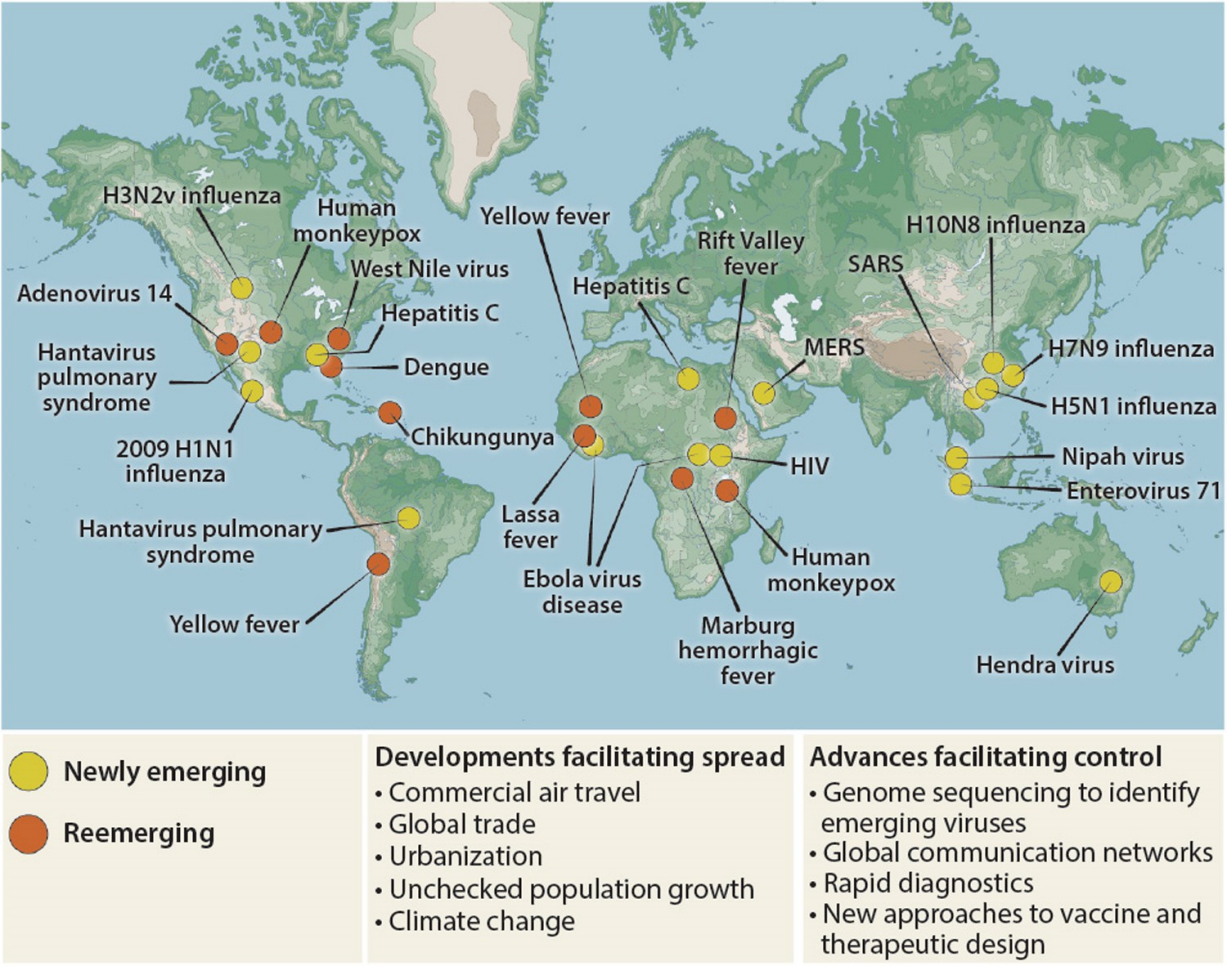
Mapping emerging viral diseases. Emerging diseases in new locations (orange) or caused by newly emerging viruses (yellow) are shown. Spread of emerging diseases are facilitated by urbanization and globalization, such as commercial air traffic and trade (with reprint permission taken from Marston and colleagues (https://www.science.org/doi/10.1126/scitranslmed.3009872); [6]).

Although many established diseases, such as tuberculosis, cholera, and malaria, have bacterial or protozoal origin, the majority of relevant newly emerging and reemerging diseases in the past century have been caused by viruses (Fig 1) [2]. They are mainly based on zoonotic events, as it occurred for HIV-1 being transmitted from chimpanzees to humans in Central Africa [3], MERS-CoV, which was transmitted from camels to humans in Arabia [4], or the emergence of the arthropod-born Zika virus, which spread from mosquitos to humans [5].

Most of the zoonotic pathogens are not well adapted to humans and only emerge sporadically through spillover events that may lead to localized outbreaks, so called “viral chatters” [7–9]. However, these spillover events increase the pandemic risk by providing the opportunity for viruses to become better adapted to new hosts and potentially cause human-to-human transmissions [7,10]. Although surveillance and awareness of personal and sanitary hygiene nowadays enhances, the risk of local outbreaks that may become pandemic remains and is associated with poverty, population density, and inadequate healthcare systems [11]. Especially high-risk pathogens like bat-borne *Henipaviruses* or Ebola virus (EBOV) are a burden to developing countries and may lead to a public health crisis based on the lack of disease awareness, missing surveillance or adequate healthcare systems [12]. Recent outbreaks of EBOV in Democratic Republic of Congo (DRC) or Guinea in the End of 2020 and the beginning of this year, respectively, were declared as “public health emergency of international concern” due to spread into areas that had not been affected before [12]. Thus, effective emergency treatment is needed to respond faster for mitigation and to control disease outbreaks [13].

## The discovery of *Henipaviruses*

The complexity of disease emergence can be highlighted by the emergence of the highly pathogenic Nipah virus (NiV) and Hendra virus (HeV). These zoonotic viruses cause fatal diseases in humans and animals and had been classified in the genus *Henipavirus* in the virus family Paramyxoviridae [14,15]. The genome of HeV and NiV consists of a single-stranded RNA molecule in negative-sense orientation surrounded by a lipid envelope [16]. Initially, HeV was recognized through a disease outbreak in 1994 in Australia, being named after the Brisbane suburb of Hendra where several horses and their trainer died from a pulmonary disease with hemorrhagic manifestations [17–20]. A second outbreak in Queensland, Australia also occurred in 1994 and affected 2 horses and 1 person. However, this event was only recognized in 1995, after the infected person died from relapsing encephalitis [19,21].

Despite NiV causing multiple outbreaks since its first identification in Sungai Nipah, it affected over 265 patients during the outbreaks in Malaysia (1998) and Singapore (1999), with 105 confirmed deaths [22,23]. Due to immediate and effective actions from the government, no further cases were reported in Malaysia and Singapore since then [22–25]. In 2001, an outbreak in Bangladesh occurred with 13 NiV-infected people; 9 of the patients died [24,26,27]. Since then, recurrent outbreaks have been detected almost every year in Bangladesh with a total of 17 outbreaks until 2015 [23]. These outbreaks were associated with a high mortality rate: From 261 identified cases, 199 individuals died [23,6,28]. Additional, locally restricted outbreaks took place in Siliguri, West Bengal, India, in 2001, with a case fatality rate of 68% [22,23,29,30] and a repeated outbreak in Nadia, West Bengal, India, in 2007, where all infected people died within 1 week after infection [22,24,27,30]. In 2014, the Philippines reported 17 confirmed NiV infections in humans; 9 patients died [31]. The latest outbreaks occurred 2018 in Kerala, India, with a case fatality rate of 91% (23 infected patients) and 2019; after 7 days of severe symptoms, the patient fully recovered [32].

## Socioeconomic burden of *Henipavirus* outbreaks

Malaysia (43%), Bangladesh (42%), and India (15%) represent all incident cases of human NiV infections worldwide [27]. Apart from the human catastrophe of high morbidity and mortality rates during documented epidemic outbreaks, the economic impact is tremendous [33]. After the first NiV outbreak in 1999, Malaysian pig industry and related sectors suffered enormous damage, i.e., 1.1 million pigs were culled costing about US$66.8 million with a total decrease in the Malaysian economy of around 30% during that time [33,34]. In addition to direct losses in the livestock sector, the feed industry and oil and fat production were most affected [32]. Compared to the economic losses resulting from the EBOV outbreak in 2014, with GDP losses of US$2.2 billion in Guinea, Liberia, and Sierra Leone in 2015 [35], the burden on the Malaysian economy appears modest. Nevertheless, the economic situation in these countries is so different that a direct comparison of the overall figures does not allow for an accurate interpretation and assessment of the impact on the country. Due to the high socioeconomic burden that NiV and HeV outbreaks cause, intervention plans had been developed in several countries, including campaigns, staff costs, pretesting of materials, field visits, and transportations. In Bangladesh, these activities increased the economic damage to a total of US$255,000 [33] and led to a decline of the economic stability in affected countries [27]. Thus, there is an urgent need for information and awareness raising, including improved contact tracing, better knowledge of transmission routes to implement appropriate hygiene measures, early diagnostics, and effective therapies to reduce the socioeconomic burden.

## Transmission of *Henipaviruses*

**Fig 2:**
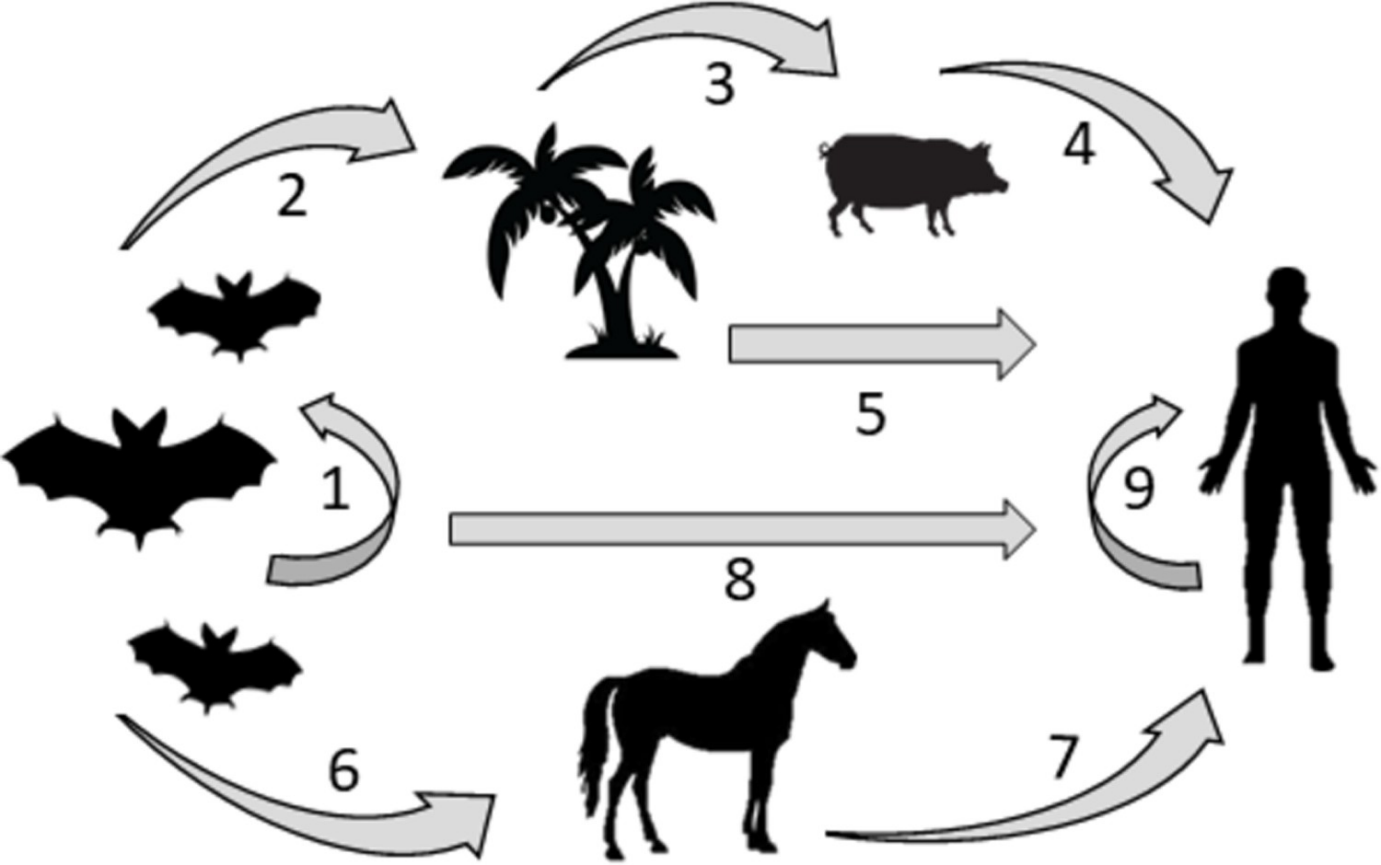
Schematic representation of pathogenic *Henipavirus* transmission from the natural host, fruit bats, to susceptible species. Shown are supposed transmission routes: (1) from bats to bats via placental transmission, lactation, or matting; (2) fruit consumption; (3) excretion and partially eaten fruits; (4) from pig to farmer (NiV Malaysia); (5) date palm consumption (NiV Bangladesh); (6) excretion; (7) from horse to owner (HeV Australia, NiV Philippines); (8) bite, scratch, etc.; and (9) from human to human (NiV Philippines, NiV Bangladesh).

For both HeV and NiV, the *Pteropus* fruit bat, also known as flying fox, is considered as the natural animal reservoirs [15,36,37]. Transmission is supposed to occur from bats via saliva, urine, and excreta to humans with pigs (NiV) or horses (HeV and NiV) as intermediate hosts **(Fig 2)**. Spillover events from bats to the intermediate hosts or humans are due to consumption of contaminated fruits or contact with contaminated secretions [29,38].

Since the first emerge of HeV in Australia, 55 events have been reported that caused fatal infections with 100 deaths in horses, mainly due to respiratory failure [36]. Seven human HeV infections are documented; 4 patients died [18,39–42]. To prevent human HeV infections, horses diagnosed positive for HeV are subsequently killed [21]. Since 2015, an equine HeV vaccine has been fully registered in Australia, and no HeV-vaccinated horse has been tested positive for HeV infection since then [21]. Nevertheless, although vaccination against HeV exist, spillover events of HeV infection in horses still occur, since uptake of the vaccine is limited due to misperceptions of horse owner, such as the underestimation of severity of HeV infection, vaccine safety or impact on the performance of (racing) horses, costs or effectiveness of the vaccine [43–45]. However, human interference into nature bears an increasing risk of expansion of flying fox populations into urban areas, resulting in direct transmissions from the viral reservoirs to humans [46].

NiV-infected pigs show symptoms that vary by age but include neurological and respiratory signs such as tremors and severe cough, also known as “barking cough” [47,48]. Spillover events from *Henipavirus*-infected pigs to slaughterhouse and farm workers possibly occur through contact with contaminated pigs and their meat during processing of infected pigs in slaughterhouses. In the NiV outbreaks of Malaysia and Singapore, infected pigs had been identified as the main source of infections [23,49]. However, in the Bangladesh outbreaks, no evidence could be found for transmission via pigs. In these cases, ingestion of date palm sap, contaminated by fruit bats secretion and excreta, are suggested to be the main source of infection [23,28,50,51].

Human encroachment into flying fox habitats, i.e., by deforestation, but also climate change increases the risk of outbreaks in new locations by changing habitats of the zoonotic reservoir hosts [14,52–54]. In addition, globalization and international trade facilitate the spread of disease, as shown in the NiV outbreaks in Singapore and Malaysia. Importing infected pigs from a contaminated area in Malaysia infected 11 slaughterhouse workers and caused one death [15,23,25]. Based on these outbreaks, a study aimed to identify the potential threat of pig trading in the transmission of NiV and examined how long-distance transportations of living pigs may facilitate disease dissemination in Thailand [55]. Findings showed that, although the risk of NiV dissemination through pig trade is low, it is not negligible and may cause local outbreaks that requires preventive strategies concerning international trading [55].

## The threat of a potential global spread

Despite transmission and dissemination of *Henipaviruses* via infected livestock, a potential threat arises from human-to-human transmission. Whereas only a few cases of human HeV infections are reported, several NiV outbreaks have included person-to-person transmission with case fatality rates of up to 70% [29,56]. The capacity for NiV to spread in hospital settings between staff and patients was shown in an outbreak 2001 in Siliguri, India, which affected 66 people. The outbreak originated from an unidentified patient admitted to Siliguri District Hospital who infected 11 people [50]. Thus, the ability of NiV to spread from patients to nursing staff has raised concern that the virus might adapt to more efficient human-to-human transmission [15,29,37,50,57–59]. In terms of the ability of human-to-human transmission, the different NiV strains differ. Therefore, it is quite conceivable that one of these strains acquires mutations during human infection that lead to more efficient and sustained human-to-human transmission. However, the virus is not dependent on replication in humans, so it can continue to spread through vectors even without adaptations to humans. In this regard, we should keep in mind the constant man-induced environmental changes, as these can lead to altered transmission patterns in emerging viruses with the chance for genetic variation. The lack of knowledge on the transmission route of the virus in the environment bears a high risk of a potential pandemic spread by facilitating viral transfer and disease transmission [29,57]. Together with considerable travel activities, including long-distance air traffic, but also increased international trading, might elevate the pandemic potential of *Henipaviruses* [29]. Thus, the perception of virus stability on surfaces under distinct environmental conditions as well as the successful inactivation of viral loads on these surfaces is a pressing need to improve safety practices for caretakers, researchers, and public health experts supporting an effective infection control [60,61].

Up to date, only few studies exist that examine *Henipavirus* stability on surfaces and objects and their role in viral disease transmission [62,63]. Fogarty and colleagues [57] analyzed the persistence of NiV and HeV under natural conditions relevant to bat transmission. The group tested viral loads of *Henipavirus* in bat urine and fruits under distinct conditions and revealed that survival of *Henipaviruses* in the environment varies between few hours and a couple of days is highly dependent on temperature and desiccation [57]. These results indicate that a short half-life of the virus requires close contact to the infected hosts or contaminated material for a successful transmission. However, under optimal conditions, *Henipavirus* is able to persist for days, which makes vehicle-borne transmission a potential source of danger [57].

Epidemiological studies of NiV outbreaks in several countries suggested that besides consumption of contaminated food, intermediate hosts and infected animals are the main source for human infections [23,28]. NiV-infected pigs are supposed to be an important factor for infections in humans. Transmission via pigs potentially occur through the respiratory route, but close contact with infected tissues of pigs might also result in NiV transmission [23,64–66]. When examining the risk for transmission that might involve bodily fluids, Smither and colleagues [67] showed that the stability of NiV in blood or cell culture media under distinct conditions can last up to 1 week at room temperature, and, hence, providing the opportunity to cause fatal infections for a longer time period.

Despite transmission of *Henipavirus* via contaminated food [68], bats, or intermediate hosts, spread from infected persons to naïve individuals is a high-risk factor. Patients infected with *Henipavirus* shed viruses in body secretions, including blood, feces, urine, or saliva [50,51]. Studies have shown that the highest risk of being infected exists for family members who provide continuous care, and also for caregivers during hospitalization [59,69–71]. Watanabe and colleagues demonstrated that NiV in human serum samples is able to survive for as long as 7 days at room temperature [72]. To analyze the risk potential of NiV-infected patients’ fomites contaminated surfaces in hospitals, samples collected in close proximity to diseased people from, i.e., the wall beside the patients’ bed, bed rail and sheets, clinical record files, and multipurpose towels were examined [70]. While no virus was detected on clinical files and wall surfaces nearby the patient, the most contaminated surfaces were bed sheets and towels [70]. However, these data did not show for how long infectious virus particles may persist on these surfaces.

Until now, limited data exist on the stability of *Henipaviruses* on surfaces. The ability to measure the persistence of NiV and HeV under different environmental conditions will therefore contribute to elucidating transmission routes, as in general studies on the survival of viruses in the environment and on surfaces and objects helps to intervene in and control viral outbreaks [73]. Based on this knowledge and the understanding on the role of surfaces on facilitating virus persistence, disinfectants can be adjusted to be more effective and drastically reduce viral titers in any spillage or contamination to limit or prevent the spread of viral infections and pathogen transmission [61,62]. After each *Henipavirus* outbreak, questions arise regarding adequate elimination and inactivation of medical waste and human remains [32]. So far, terminal decontamination at the end of outbreaks are an important challenge as no defined standards and guidelines are currently available [32]. After the Kerala outbreaks safety protocols came up that include using 2% to 5% Lysol/5% to 10% freshly prepared household bleach, followed by autoclaving or incineration. However, developing countries cannot afford expensive equipment and therefore need inactivation methods that are adapted to the possibilities without having to make any concessions in terms of security [27,32]. There are no studies performed to investigate the survival time of the pathogen on disinfected surfaces and objects or in human dead bodies [27].

## Clinical features of *Henipavirus* infections

Once infected with *Henipaviruses*, the incubation period ranges from a few days to about 2 months depending on the route of transmission [17,74,75]. While the median incubation period in case of raw date palm sap consumption was 10 days, exposure to infected pigs can result in incubation periods of up to several weeks, whereby the majority of patients show symptoms after 2 weeks or less [24,75,76]. In humans, HeV infections result in most cases in influenza-like symptoms such as fever, myalgia, headaches, cough, and pharyngitis, before patients develop a fatal encephalitis [15,27]. Individuals infected with NiV typically present with clinical symptoms often associated with neurological disorders and acute encephalitis, while in addition, respiratory symptoms are found in approximately 25% of all patients [77]. Person-to-person transmission of viral particles is thought to occur at late stages of disease progression in NiV- and HeV-infected patients when the respiratory tract is involved in pathogenicity [50,78,79]. In fact, during the 2018 outbreak in Kerala, India, all nosocomial transmissions potentially occurred through droplet infection while the index patient was near end-stage disease and had a persistent cough [27,79,80]. This outbreak stresses the awareness among public and health caretakers for effective containment measures to prevent future outbreaks [32]. Precautions by safety measures such as personal protective equipment and proper hygiene after handling infected patients are important as rapid isolation and minimizing patient-to-caretaker exposure via bodily fluids [27,32]. Hence, the urgent need for a substantiated knowledge exists about the persistence of viruses outside their vectors or infected hosts to reduce the risk of further spread of the disease [62,63].

Currently, there is no vaccine available and treatment of patients infected with *Henipaviruses* is primarily based on supportive care [81,82]. Thereby, raising the awareness of risk factors, prevention of transmission, and controlling outbreaks by trained healthcare workers is the only effective principal measure, so far.

## Closing remarks

The recent SARS-CoV-2 pandemic has shown limitation of disease containments in a globalized world. Within months, we went from the first case of COVID-19 to thousands of deaths reported worldwide [83]. This pandemic has raised concerns about effective measurements and strategies to prevent the global spread of diseases. International air traffic, traveling, and international trading induce higher risks during disease outbreaks and hamper real-time monitoring and identification of infected people by health authorities [83]. Disease outbreaks, including the NiV outbreak in India in 2018, the Lassa virus outbreak in Nigeria in 2018, or the reemergence of Ebola in Guinea and the DRC in 2021, raised the question how to predict outbreaks and develop response plans to be able to manage and control spread of diseases [84]. In addition, there is a continuing risk from newly discovered *Henipaviruses* and Henipa-like viruses of endemic and epidemic potential in the human population. In 2009, a study contacted in Kumasi/Ghana found putative *Henipaviruses* via RNA analysis of fecal material from African straw-colored fruit bats and discussed the probability of a fecal–oral transmission in comparison to more likely transmission routes like the consumption of bat meat [85]. In 2012, the isolation of a novel paramyxovirus, named Cedar virus (CedPV), from pooled urine samples of fruit bats in Cedar Grove, South East Queensland, Australia, was reported [86]. Though initial studies revealed CedPV being nonpathogenic in *Henipavirus* infection models, an elevated IFN-b induction by CedPV compared to HeV in human cells [86].

Effective precaution and containment measures presuppose a knowledge at all levels of disease emergence, i.e., understanding the route of transmission, stability outside vectors and hosts on objects and surfaces, rapid diagnosis, and an effective treatment. Therefore, gaining a deeper understanding of the molecular mechanisms of replication in host cells and the persistence of pathogens in the environment are fundamental to protect against infectious diseases with epidemic and pandemic potential. Due to the drastic impact of zoonotic diseases and often high mortality rates, it is recommended that scientists, public health authorities, and policy makers pay attention to the pandemic risk of *Henipaviruses*.

**Key Learning Points**

> *Henipaviruses* transmit via distinct infection routes including contact to contaminated food or meat or direct contact to infected animals or persons.
> Personal protective equipment and proper hygiene are highly recommended for farm and slaughterhouse workers as well as healthcare workers and medical personnel.
> To date, there is no vaccine available leaving the treatment of patients infected with *Henipaviruses* primarily to the application of supportive care.

**Top Five Papers**

1. Pillai VS, Krishna G, Veettil MV. Nipah Virus: Past Outbreaks and Future Containment. Viruses. 2020;12(4).
2. Daszak P, Zambrana-Torrelio C, Bogich TL, Fernandez M, Epstein JH, Murray KA, et al. Interdisciplinary approaches to understanding disease emergence: the past, present, and future drivers of Nipah virus emergence. Proc Natl Acad Sci U S A. 2013;110(Suppl 1):3681–8.
3. Yuen KY, Fraser NS, Henning J, Halpin K, Gibson JS, Betzien L, et al. Hendra virus: Epidemiology dynamics in relation to climate change, diagnostic tests and control measures. One Health. 2021;12:100207.
4. Hassan MZ, Sazzad HMS, Luby SP, Sturm-Ramirez K, Bhuiyan MU, Rahman MZ, et al. Nipah Virus Contamination of Hospital Surfaces during Outbreaks, Bangladesh, 2013–2014. Emerg Infect Dis. 2018;24(1):15–21.
5. Epstein JH, Field HE, Luby S, Pulliam JRC, Daszak P. Nipah virus: impact, origins, and causes of emergence. Curr Infect Dis Rep. 2006;8(1):59–65.
